# Case Report: Exaggerated estradiol secretion in an infant with hypothalamic hamartoma

**DOI:** 10.3389/fendo.2025.1598734

**Published:** 2025-09-25

**Authors:** Maja Vinkovic, Duje Braovac, Katja Dumic Kubat, Ivan Jovanovic, Maja Banovic, Nevena Krnic

**Affiliations:** ^1^ Department of Pediatrics, University Hospital Centre Zagreb, Zagreb, Croatia; ^2^ School of Medicine, University of Zagreb, Zagreb, Croatia; ^3^ Department of Radiology, University Hospital Centre Zagreb, Zagreb, Croatia; ^4^ Department of Gynaecology and Obstetrics, University Hospital Centre Zagreb, Zagreb, Croatia

**Keywords:** hypothalamic hamartoma, central precocious puberty, estradiol, mini-puberty, case report

## Abstract

**Objectives:**

Hypothalamic hamartoma (HH) is an important cause of central precocious puberty (CPP) in young children but is rarely described in infants. Interpretation of laboratory data could be difficult because gonadotropins and estradiol levels often overlap in healthy infants with mini-puberty and children with HH. Extremely elevated estradiol levels are mostly described in girls with peripheral precocious puberty.

**Case presentation:**

We present a 5.5-month-old girl with vaginal bleeding, significantly elevated estradiol levels (up to 3,974 pmol/L), elevated gonadotropins, and right ovarian cyst. Laboratory and radiologic evaluation revealed the HH as a cause of CPP. Immediately after the start of treatment with depot gonadotropin-releasing hormone analogue, age-appropriate undetectable levels of estradiol were achieved, with ovarian cyst regression and cessation of pubertal changes.

**Conclusion:**

If observed in the period of mini-puberty, high levels of estradiol accompanied by unsuppressed gonadotropins can complicate the discrimination between central and peripheral precocious puberty. This challenge emerges particularly due to the absence of the negative feedback mechanism in children with HH. This is the first report identifying extremely high estradiol levels as part of the phenotypic spectrum of HH in infants.

## 1 Introduction

Pubertal changes in female infants are most observed as part of benign premature telarche, which rarely requires further laboratory evaluation (1). However, central or peripheral precocious puberty can also occur, although central precocious puberty in infants is extremely rare (1, 2).

Hypothalamic hamartoma (HH) is a well-recognized cause of central precocious puberty (CPP), particularly in very young children, with an average age of initial symptoms of around 1.5 years (3, 4). Estradiol levels in healthy girls and those with CPP are usually only slightly elevated in this age group. In contrast, significant elevation has been described in premature infants or girls with ovarian tumors or functional cysts (5–8).

We present a 5.5-month-old female infant who presented with vaginal bleeding and was found to have extremely elevated estradiol levels and CPP caused by HH.

## 2 Case presentation

**Figure 1:**
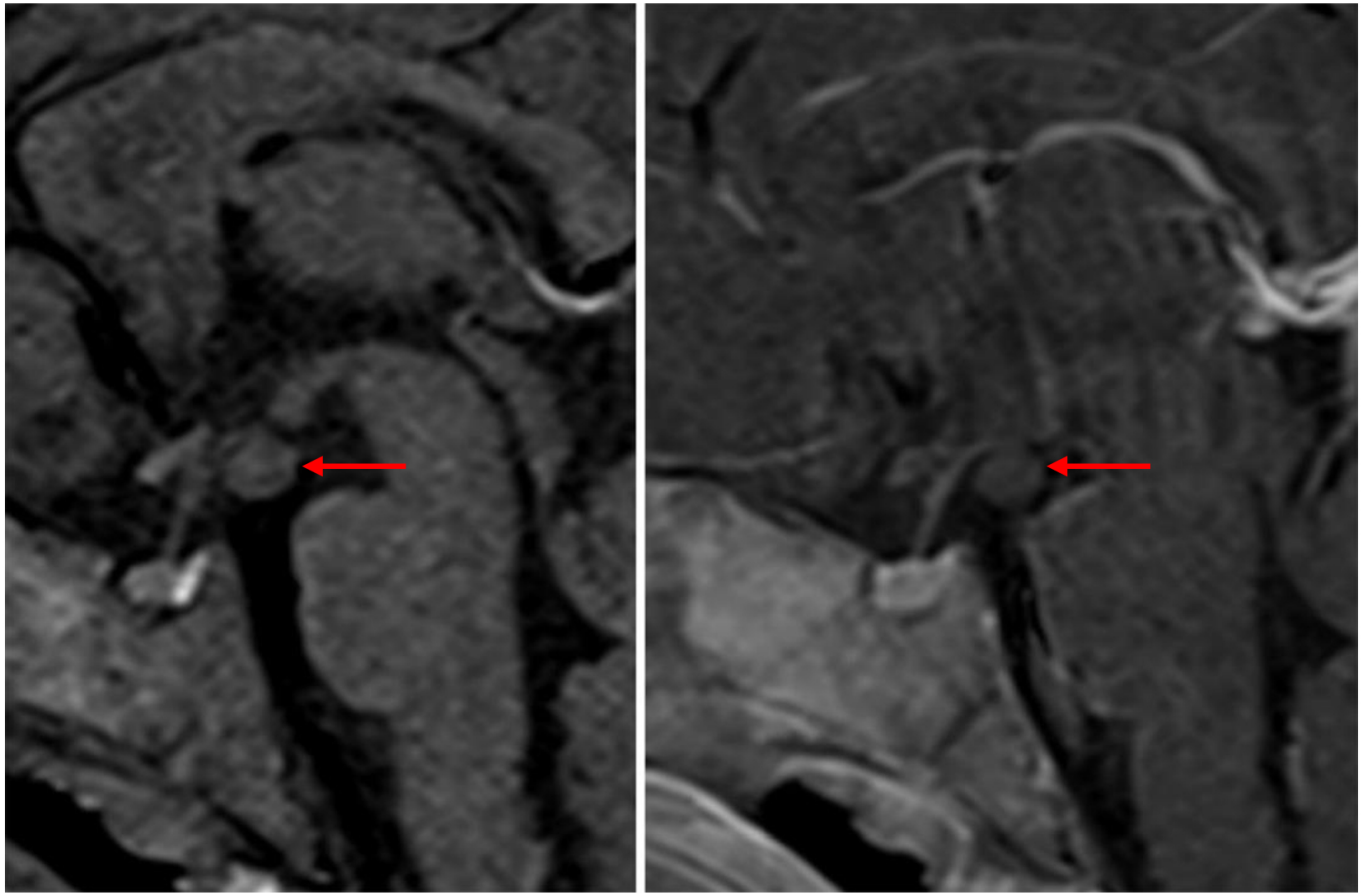
T1-weighted sagittal MR images in a 5.5-month-old infant with central precocious puberty and exaggerated estradiol secretion before and after gadolinium administration demonstrated a hypothalamic hamartoma. A rounded mass is located below the floor of the third ventricle, projecting into the suprasellar cistern behind the infundibulum of the pituitary gland. It is isointense to grey matter on T1 weighted images and does not enhance after gadolinium administration.

A 5.5-month female infant was referred for further evaluation due to prolonged vaginal bleeding. She is the third child of healthy, unrelated parents, born from an uneventful pregnancy at 38 + 2 weeks of gestation (birth weight 3,380 g, + 0.37 SDS; length 51 cm, +0.17 SDS). Her previous medical history was unremarkable, and developmental milestones were age appropriate. There were no data on previous infection, trauma, topical use of estrogen-containing cosmetic products, or sexual precocity in family members. Bloody vaginal discharge resolved spontaneously after 5 days. On physical examination, her height and weight were 1.19 SDS and 0.31 SDS, respectively. The breast development was Tanner stage II, with enlarged, normally pigmented nipples and no pubic or axillary hair. There were no caffe-au-lait spots, but large light beige discoloration with irregular margins, covering the left abdominal area, up to the left rib cage, was noted. Endocrine laboratory evaluation showed elevated basal gonadotropins with markedly elevated estradiol levels, measured during, and following the resolution of vaginal bleeding (**Table 1**). Serum tumor markers (α-fetoprotein, β-subunit human chorionic gonadotropin, carcinoembryonic antigen, cancer antigen 125, carbohydrate antigen 19–9) and thyroid function tests (free thyroxine, thyroid-stimulating hormone) were normal. Pelvic ultrasound revealed a slightly enlarged uterus measuring 1.96 × 1.45 × 3.09 cm with an endometrial thickness of 3.1 mm. No follicles, cysts, or tumors were detected in the ovaries (right ovary 1.58 × 0.82 cm; left ovary 1.54 × 1.14 cm). Because of extremely high estradiol levels, autonomous ovarian secretion was also considered, so both brain and pelvic MRIs were performed on the 24^th^ day after bleeding. Estradiol levels, measured simultaneously in two different laboratories, were about four times higher than initial (**Table 1**). Pelvic MRI revealed a cyst in the right ovary measuring 17 mm, and repeated pelvic ultrasound also described an avascular right ovarian cyst 20 × 14 mm. However, a brain MRI confirmed the diagnosis of HH (isointense peduncular lesion originating from mammillary bodies, 6 mm in diameter) (**Figure 1**).

**Table 1:** Laboratory data of a female infant with central precocious puberty caused by hypothalamic hamartoma.

After vaginal bleeding	4^th^ day	9^th^ day	24^th^ day	2 months^c^	3 months^c^	SI units
LH	1.9	–	4.6	0.6	0.6	IU/L
FSH	4.4	–	2.4	2.6	2.5	IU/L
Estradiol^a^	830	520	2,340 3,974^b^	<37	<37	pmol/L

LH, luteinizing hormone; FSH< follicle-stimulating hormone, ^a^levels measured using immunoassay method, ^b^measured in different laboratories, ^c^levels measured after initiation of treatment with depot gonadotropin-releasing hormone analogue.

Diagnosis of central precocious puberty due to HH was made, and the treatment with monthly gonadotropin-releasing hormone analogue was commenced. One month after the treatment initiation, the estradiol levels were undetectable and the cyst in the right ovary subsided completely.

On follow-up, 4 months after treatment initiation, gradual breast tissue regression was noticed, and estradiol levels remained low. The developmental milestones are in the normal range, and no seizures are noticed.

Written informed consent was obtained from the proband’s parents, and Institutional Ethical Board approval for publication of data was acquired.

## 3 Discussion

The hypothalamic–pituitary–gonadal axis (HPGA) is active in infancy, leading to cyclic pituitary and gonadal secretion characterized as mini-puberty (1). During mini-puberty, the levels of luteinizing hormone (LH), follicle-stimulating hormone, and estradiol reach the pubertal range, but besides breast tissue enlargement, the development of other visible changes is rare (9). Vaginal bleeding is seldom described as a manifestation of exaggerated mini-puberty in premature infants (7). If there is a progression of clinical symptoms or more severe pubertal changes than age-appropriate are present, it is important to investigate potential underlying pathological causes.

Diagnosing precocious puberty in infants is often challenging due to difficulties interpreting laboratory data. Basal levels of LH in CPP and healthy infants with mini-puberty are indistinguishable. The response to gonadotropin-releasing analogue stimulation testing may be more pronounced during infancy, and the results have not been well established, leading to overlap between CPP and benign forms of precocious puberty in infants (1).

Estradiol levels during mini-puberty in healthy infants reach up to 100 pmol/L (5); however, much higher estradiol levels were reported in preterm infants as exaggerated mini-puberty manifestation. Extreme forms of mini-puberty have been reported exclusively in premature infants. It is speculated that this phenomenon could present an adaptive mechanism that promotes linear growth and maturation of reproductive organs and target tissues or indicate an immaturity of negative feedback control of HPGA (7, 10). All reported patients with exaggerated mini-puberty presented with vaginal bleeding, elevated levels of gonadotropins and estradiol. Most of them had ovarian cysts on pelvic ultrasound examinations and normal findings on brain MRI (7). High estradiol levels in preterm infants are also reported in ovarian hyperstimulation syndrome (11). Pituitary activity during infancy can rarely lead to ovarian hyperstimulation with the formation of functional ovarian cysts. Ovarian hyperstimulation was also described in gonadotroph adenomas (only two reported in children), with estradiol levels as high as 31,731 pmol/L and multicystic enlarged ovaries (12).

In a girl presenting with central precocious puberty at an early age, it is mandatory to exclude HH. According to the presentation on MRI scans, the HH can be classified as either pedunculated or sessile, with CPP being more common in the former (4). There are several plausible explanations of the HH effect on pubertal development. These include the activation of normal hypothalamic tissue due to compression, anatomical communication and secretion of paracrine factors, or autonomic pulsatile release of gonadotropin-release hormone from HH (4). The sequence of pubertal changes and levels of gonadotropins and sex hormones in children with HH are similar to normal puberty. The use of gonadotropin-releasing hormone agonists is the first-line treatment of CPP in children with HH (13).

Among infants with HH, basal levels of gonadotropins and estradiol are usually in the normal pubertal range (14). Higher than normal levels of estradiol are rarely reported in infants with HH (15). Our patient presented with extremely elevated estradiol levels, with cyclical increased levels mimicking the normal menstrual cycle. However, gonadotropin levels were not suppressed, which supported the diagnosis of CPP. Such high estradiol levels are usually described only in patients with peripheral precocious puberty, where excessive hormone secretion originates from either gonads or adrenal glands, while gonadotropin levels are suppressed (14). Excessively high estradiol levels were described in prepubertal girls with ovarian steroid cell tumors (estradiol levels ranging from 348.8 to 2,420.9 pmol/L) (6). Elevated levels of estradiol (680 pmol/L) and unsuppressed gonadotropin levels were also reported by Feilberg Jørgensen et al. in an 18-month girl with HH and juvenile granulosa cell tumor. The authors speculated that continuous hormonal stimulation from an early age by HH might have contributed to ovarian tumor development (8). However, to the best of our knowledge, there is no report of such excessive estradiol secretion caused exclusively by HH and suppressed immediately with gonadotropin-releasing hormone analogue treatment.

Our patient had supranormal estradiol levels (5–39 times above the upper range for healthy female infants), but despite this, gonadotropin levels were not suppressed. Our results align with the patient described by Feilberg Jørgensen et al. and illustrate the loss of negative feedback mechanism in HH (8).

We can speculate that our patient, in addition to HH, may also have endogenous ovarian dysfunction leading to a propensity for ovarian cyst formation and high estradiol level production, similar to ovarian hyperstimulation syndrome. However, due to rarity of HH finding in infancy, further studies should confirm if such high estradiol levels are incidental findings or are characteristic pattern of secretion in immature hyperstimulated ovaries of young infants.

With our case report presentation, we would like to emphasize that even extreme estradiol levels can be a phenotypic spectrum of HH in infants and that interpreting laboratory data of infant with CPP can pose difficulties in this age group.

## Data Availability

The original contributions presented in the study are included in the article/Supplementary Material. Further inquiries can be directed to the corresponding author.
